# High impact of sleeping problems on quality of life in transgender individuals: A cross-sectional multicenter study

**DOI:** 10.1371/journal.pone.0171640

**Published:** 2017-02-15

**Authors:** Matthias K. Auer, Anita Liedl, Johannes Fuss, Timo Nieder, Peer Briken, Günter K. Stalla, Thomas Hildebrandt, Sarah V. Biedermann, Caroline Sievers

**Affiliations:** 1 Research Group Clinical Neuroendocrinology, Max Planck Institute of Psychiatry, Munich, Germany; 2 Institute for Sex Research and Forensic Psychiatry, University Medical Center Hamburg-Eppendorf, Hamburg, Germany; 3 Department of Gynecology and Obstetrics, Erlangen University Hospital, Erlangen, Germany; 4 Clinic of Psychiatry and Psychotherapy, Center for Psychosocial Medicine, University Medical Center Hamburg-Eppendorf, Hamburg, Germany; Public Library of Science, FRANCE

## Abstract

**Introduction:**

Studies in the general population suggest that determinants of QoL are often sex-dependent. Sex-dependent analyses of QoL in transgender populations have not been performed so far.

**Aim:**

To identify sex-specific and potentially modifiable determinants of QoL in transgender patients

**Methods:**

In this cross-sectional multicentre study including 82 transwomen (TW) and 72 transmen (TM) at different treatment stages, we investigated potential determinants for QoL focusing on the impact of mood (BDI, STAI-X), sleep quality (PSQI), chronic pain (GPQ), body image (FBeK) and social support (SSS).

**Main outcome measure:**

Health-related quality of life measured with the Short Form (36) Health Survey (SF-36).

**Results:**

The age-adjusted SF-36 total score and its subscales did not significantly differ between TM and TW. Using a multivariate regression analysis approach, we identified common but also sex-dependent determinants for QoL (Adjusted R^2^ = 0.228; 0.650 respectively). Accounting for general characteristics such as age, BMI and treatment status, sleep quality according to the PSQI was an independent and strong determinant of QoL in both sexes (β = -0.451, p = 0.003 TM; β = -0.320; p = 0.0029 TW). Chronic pain was a significant independent predictor of QoL in TM (β = -0.298; p = 0.042) but not in TW. In contrast, anxiety (β = -0.451; p< 0.001) being unemployed (β = -0.206; p = 0.020) and insecurity about the own appearance (FBeK) (β = -0.261; p = 0.01) were independent predictors of QoL in TW. The rate of those reporting high sleep disturbances (PSQI ≥5) was high with 79.2% in TW and 81.2% in TM. Accordingly, age-adjusted QoL was also significantly lower in those reporting poor sleep in both sexes.

**Conclusions:**

Sleep strongly affected QoL in both genders, while other factors, like pain and body image, seem to be gender specific in transgender individuals.

## Introduction

Gender Incongruence (GI) is characterized by a strong gender identification that is incongruent with the sex assigned at birth. GI often results in significant gender dysphoria (GD) emerging from the experienced incongruence [1]. The etiology of both GI/GD is still far from being understood. However, some recent theories highlight biological factors associated with GI/GD [2, 3]. The primary aim of transgender care lies in overcoming the individual’s GD by homogenizing gender identity with the phenotypic sex by–amongst others–sex hormone treatment (SHT) and transition-related surgeries (TRS). Even though the outcome of those medical interventions can be judged according to functional and aesthetic criteria, one of the overarching objectives of any single intervention within transgender care should be the improvement of a person’s health-related quality of life (HrQoL) [4]. Determinants of QoL are however highly subjective and also depend on the cultural and social context [5]. In addition to the medical outcome, many transgender individuals are still struggling with familial and social difficulties that may affect their QoL [6]. Facing these difficulties, while struggling with GI/GD represents a huge burden for these patients. This may partly explain for the high rates of mental health problems [7, 8]. In line, several studies have reported that QoL in transgender individuals is impaired in comparison to birth-sex-matched controls [9–13]. Studies investigating determinants of QoL in transgender individuals have primarily focused on specific transition-related interventions such as SHT and TRS although some studies have also included socioeconomic factors into their analysis [10, 14]. Most studies report that endocrine [15–17], and surgical treatments [9–11] are improving QoL. Further factors affecting QoL include having a partner [10, 11, 18], the extent of family support, as well as employment status [17]. We have shown previously in cohorts of patients with pituitary adenomas that potentially modifiable factors such as comorbid anxiety or depressive disorders [19], pain [20] and also sleep quality [21] may be more powerful to predict QoL than “somatic” factors, such as adequate hormone treatment or in the present case, the aesthetic and functional outcome of TRS. In line, depressive symptoms have been shown to be an independent predictor for a lower QoL [15] in transgender individuals. Little attention has been paid so far to the fact that sex itself can be a mediating factor for determinants of QoL [22, 23], though studies have shown that QoL between transmen (TM) and transwomen (TW) may significantly differ [10, 14].

We therefore hypothesized that different factors may predict QoL in TM and TW. This would offer the chance for gender-specific health care interventions. We included socioeconomic factors that have been shown to affect QoL before [15, 17]. Persons with GD receive regular psychotherapeutic treatment before and during their transition in many countries including Germany. Therefore, it is of particular clinical importance to find modifiable factors influencing QoL. This should help to improve treatment strategies specifically for this clinical population and thus ultimately improve QoL.

### Main outcome measures

The aim of our study was to identify factors that could be addressed by specific interventions in a health care setting to further improve QoL in transgender individuals.

## Materials and methods

### Sample

A total of 154 transgender individuals (82 TW, 72 TM) were included in this study. All participants were part of an observational multicenter study in Germany to assess the effects of medical interventions on psychological and metabolic outcomes of transition-related health care. This study reports on data that had been collected between November 2013 and July 2015 at four different centers: the Department of Endocrinology of the Max Planck Institute of Psychiatry, Munich in conjunction with the “Hormon- und Stoffwechselzentrum München”, Munich, the Gynaecological Department of the University hospital of Erlangen, the Interdisciplinary Transgender Health Care Center with the Institute for Sex Research and Forensic Psychiatry, Hamburg. All patients were diagnosed with gender dysphoria (DSM-5, 302.85) or Transsexualism (ICD-10, F64.0) and were treated according to the 7^th^ version of the Standards of Care (SoC 7) published by the World Professional Association for Transgender Health [24],although there are some national peculiarities due to health insurance´s reimbursement policies. Every eligible subject was asked for participation during a routine visit at the corresponding center. Individuals treated at the Interdisciplinary Transgender Health Care Center associated with the Institute for Sex Research in Hamburg were included right after referral for SHT, while participants from other centers were included before and after initiation of SHT.

The study was approved by the local ethics committees of the Ludwig Maximilian University of Munich, the Friedrich-Alexander University Erlangen-Nürnberg and the regional physician chambers (Landesärztekammern) of Bavaria and Hamburg. The study was conducted in accordance with the ethical standards laid down in the 1964 Declaration of Helsinki. All participants gave written informed consent. This study is registered at clinicaltrials.gov (identifier: NCT02185274).

### Data acquisition

The following variables were acquired by a self-administered questionnaire and verified with the data available in the individuals clinical records: Age, sex, age of onset (early vs. late onset), educational level (not having graduated, low, intermediate or high education and holding a university degree), employment status, self-estimation of the financial situation (good, average, bad), relationship status (single vs. in a relationship), the current use of SHT and having undergone one or more TRS. Participants being classified as having undergone any genital surgery had per our definition undergone orchiectomy, penectomy and vaginoplasty if they were transitioning from a male to a female physical appearance and ovariectomy, hysterectomy with or without phalloplasty if they were transitioning from a female to a male physical appearance. Additionally, we report on the frequency of performed mastectomies and breast augmentation surgeries. Participants were further asked to report if they had suffered from any kind of pain that had lasted for more than 5 consecutive days in the last 3 months (chronic pain). In this case, they were further asked to fill out the German pain questionnaire (GPQ) version 1. They also completed a self-constructed questionnaire rating potential side effects of hormonal therapy such as flushing, sweating, acne etc. with a rating from 0 (not at all) to 4 (severe).

### Instruments used

#### The 36-item short-form (SF-36)

The SF-36 consists of 36 items and determines health-related quality of life. The SF-36 includes one multi-item scale that assesses eight health concepts, namely vitality, physical functioning, bodily pain, general health perceptions, physical role functioning, emotional role functioning, social role functioning and mental health. A mental and physical health sum score as well as a global score can be calculated [19]. The domain scores range from 0 to 100 with higher values indicating a better subjective health status [25].

#### Beck Depression Inventory II (BDI-II)

The Beck Depression Inventory (BDI)-II is an instrument for measuring symptoms of depression. It comprises 21 questions about how the patient has been feeling in the last two weeks. A total score of 0–9 indicates minimal, a score of 10–18 mild, a score of 19–29 moderate and a score of 30–63 severe depression [26].

#### Social Support Scale (SSS)

Social support was assessed by means of the well-established Social Support Scale (F-SozU). The short version of this test consists of 22 items and measures a composite of the patients' perceived emotional and practical support as well as perception of social integration [27].

#### Fragebogen zur Beurteilung des eigenen Körpers (FBeK)

The FBeK (engl: Body Image Measure) questionnaire is a German questionnaire for assessing peoples’ subjective views of their own bodies. It contains 52 Items as the basis for the calculation of the sum scores ‘attractiveness/self-confidence’, ‘accentuation of physical appearance’, ‘uncertainty and anxiety in relation to the appearance’ and ‘physical reactions and physical–sexual discomfort’ [28].

#### State-Trait Anxiety Inventory Form X (STAI-X)

The STAI-X is a psychological inventory consisting of 40 questions on a self-report basis. The STAI-X measures two types of anxiety: (i) state anxiety or anxiety about an event and (ii) trait anxiety or anxiety level as a personal characteristic. Higher scores indicate more anxiety [29].

#### Pittsburgh Sleep Quality Index (PSQI)

Sleep quality was assessed with PSQI, an established international measure of sleep quality. The PSQI consists of 19 items, relates to the last 1-month time interval. It generates an overall score and seven component scores. In this study, we concentrated on the global score. The global score has a range of 0–21 points with a higher number of points indicating poorer sleep quality [30].

#### German pain questionnaire

The GPQ comprises demographic data, phenotypic characteristics, associated symptoms, affective and sensory qualities of pain, pain-relieving and -intensifying factors [31]. In this study we concentrated on the description of pain locations.

### Statistical analysis

Statistical analysis was performed using PASW Statistics (formerly SPSS) Version 22.0 for Windows. Sample characteristics were compared using χ2 tests and Fisher's exact test for categorical variables and the Mann-Whitney U test for ordinal and nominal variables. Univariate correlation analyses were performed using Pearson's or Spearman correlation coefficient as appropriate. Separate multivariate regression analyses were used to identify independent contributors for QoL in both sexes. Severely skewed variables were log-transformed before further analysis. A two-sided *P* value of < 0.05 was considered statistically significant.

## Results

### Sample description

General characteristics are reported in Table 1. TM and TW did only differ in terms of mean age at study inclusion (32.4 ±11.2 years vs. 42.2 ±12.4: p = 0.001). There was in particular no difference in medical treatment characteristics and socioeconomic variables such as relationship status or financial situation.

**Table 1:** General characteristics.

	Transwomen			Transmen				
	N	%		N	%			p
**Total**	82			72				
	**Mean**	**SD**			**Mean**	**SD**		
Age	42.4	12.4			32.4	11.2		**< 0.001**
BMI	24.6	3.9			25.1	4.5		n.s.
	**N**	**%**		**N**	**%**			
**Hormone treatment**								
Yes	65	79.3		58	80.6			n.s.
No	17	20.7		14	19.4			
**Estradiol**								
Gel	21	30.8		**NA**				
Tablets	21	30.8		**NA**				
Patches	24	36.9		**NA**				
Tablets + Gel		1.5		**NA**				
Cyproterone acetate		46.2		**NA**				
**Testosterone**								
Gel	**NA**			26	44.8			
Enanthate injections	**NA**			15	25.9			
Undecanoate injections	**NA**			14	24.1			
Not documented	**NA**			3	5.2			
	**Mean**	**SD**			**Mean**	**SD**		
**Time since start of hormone therapy (months)**	26	6	173		18	6	245	n.s.
**Living alone**	**N**	**%**		**N**	**%**			
Yes	51	62.2		42	58.3			n.s.
No	30	36.6		29	40.3			
Missing	1	1.2		1	1.4			
**Education**								
No graduation	4	4.9		0	0.0			n.s.
Low	19	23.2		16	22.2			
Intermediate	29	35.4		38	52.8			
High	13	15.9		6	8.3			
University	16	19.5		12	16.7			
Missing	1	1.2		0	0.0			
**Unemployed**								
Employed	74.0	90.2		67	93.1			n.s.
Unemployed	8.0	9.8		5	6.9			
**Estimated financial situation**								
Good	42.0	51.2		35	48.6			n.s.
Average	13.0	15.9		9	12.5			
Bad	26.0	31.7		27	37.5			
Missing	1.0	1.2		1	1.4			
**Age of onset**								
Early	62.0	75.6		62	86.1			n.s.
Late	20.0	24.4		9	12.5			
Missing	0.0	0.0		1	1.4			
**Surgeries**								
**Mastectomy**								
Yes	**NA**			31	43.1			
No	**NA**			41	56.9			
**Any genital surgery**								n.s.
Yes	25.0	30.5		20	27.8			
No	57.0	69.5		52	72.2			
**Penoid**								
Yes	NA			8	11.1			
No	**NA**			64	88.9			
**Breast augmentation**								
Yes	14	17.1		**NA**				
No	68	82.9		**NA**				

Comparisons were done by students’ T-test for continuous variables and X^2^ or Fishers exact test for categorical variables

Bold numbers indicate significant differences

NA: not applicable

n.s.: not significant

### Questionnaire data

Results of the different questionnaire instruments are reported in Table 2. TM and TW did not significantly differ in QoL according to the age-adjusted SF-36 global score or the different subscales. There were also no significant differences in the STAI-X total score or the BDI-II. We did not observe any difference in sleep quality assessed by the PSQI. TW reported on significantly less practical support (p = 0.02), social integration (p = 0.01) and satisfaction (p = 0.022) than TM in the SSS. They also reported on more “physical reactions and physical–sexual discomfort” according to the FBeK (p = 0.039).

**Table 2:** Questionnaire data.

	Transwomen				Transmen				
	Mean	SD	Min	Max	Mean	SD	Min	Max	P
**SF-36 global**	75.7	16.9	31.9	100.0	77.7	14.5	30.2	95.1	n.s.
Physical Functioning	91.1	10.6	55.0	100.0	92.8	11.7	40.0	100.0	n.s.
Role Limitations due to Physical Problems	77.9	34.4	0.0	100.0	84.7	30.7	0.0	100.0	n.s.
General Health Perceptions	77.3	27.0	25.0	100.0	78.1	30.2	30.0	100.0	n.s.
Vitality	55.6	20.4	10.0	100.0	56.4	19.4	0.0	90.0	n.s.
Social Functioning	80.6	22.9	12.5	100.0	82.8	23.0	12.5	100.0	n.s.
Role Limitations due to Emotional Problems	75.2	35.0	0.0	100.0	80.3	32.2	0.0	100.0	n.s.
General Mental Health	70.3	21.0	14.5	100.0	72.7	19.0	16.0	95.5	n.s.
Body pain	77.8	26.5	0.0	100.0	82.5	22.3	22.5	100.0	n.s.
									
**STAI-X total score**	92.8	22.4	59.0	155.0	94.9	20.4	64.0	140.0	n.s.
**BDI II Total score**	8.8	8.8	0.0	37.0	7.9	8.7	0.0	38.0	n.s.
**PSQI Global score**	7.0	3.5	1.0	19.0	7.4	3.4	2.0	18.0	n.s.
	**N**	**%**			**N**	**%**			
** >5** *(indicating poor sleep)*	61	79.2			13	81.2%			n.s.
									
**<5** *(indicating good sleep)*	16	20.8			56	18.8			n.s.
Missing									
**Chronic pain**	18	22.0			20	27.8			n.s
**SSS**									
Emotional support	64.4	11.6	26.0	80.0	65.7	10.9	36.0	80.0	n.s.
Practical support	32.1	7.4	10.0	45.0	35.2	7.1	14.0	45.0	**0.02**
Social integration	42.8	8.2	25.0	61.0	45.8	7.5	23.0	59.0	**0.01**
Social burden	26.1	9.1	12	54	25.6	10.5	12	57	n.s.
Reciprocity	15.0	2.8	7.0	20.0	15.8	2.7	7.0	20.0	n.s.
Satisfaction	16.3	5.0	5.0	25.0	17.8	3.9	10.0	25.0	**0.022**
Available person of trust	17.0	3.8	4.0	20.0	17.4	3.3	8.0	20.0	n.s.
**FBeK**									
Scale 1: Attractiveness/self-confidence	7.5	4.2	0.0	15.0	5.5	4.0	0.0	15.0	0.066
Scale 2: Accentuation of physical appearance	7.6	2.0	3.0	12.0	7.1	2.5	0.0	10.0	n.s.
Scale 3: Uncertainty and anxiety in relation to the appearance	4.0	2.7	0.0	11.0	4.4	2.6	0.0	12.0	n.s.
Scale 4: Physical reactions and physical–sexual discomfort	3.2	1.8	0	6	2.9	1.6	0	6	**0.039**

Compared by ANCOVA adjusted for age

Bold numbers indicate significant differences

n.s. not significant

### Contributors to QoL

#### Univariate analysis

As an explorative analysis we performed a univariate analysis with regard to potential influential factors for the SF-36 global score as a measure of QoL (Table 3). We performed a separate analysis for TM and TW as gender-specific predictors of QoL have been reported [22, 23]. In TW and TM, the STAI-X score showed an inverse correlation with the SF-36 score (r = -0.699; p < 0.001 for TW; -0.453; p < 0.001 for TM). The same was true for the BDI (r = -0.626; p < 0.001 for TW; -0.423; p < 0.001 for TM). The result of the PSQI (r = -0.622; p<0.001 TW, r = -0.530; p<0.001 TM) and suffering from chronic pain symptoms (r = -0.248; p = 0.036 TW; r = -0.355; p = 0.004 TM) were also negatively correlated with the SF-36 in both sexes. Three domains of the F-SozU were significantly correlated with QoL in TW (Social integration: r = 373; p = 001; Social burden: r = -0.319; p = 0.008; Satisfaction r = 0.375; p = 0.001) while this was not the case in any of the social support domains in TM. Again, three components of the FBeK were significantly correlated with QoL in TW (Scale 1: r = 0.304; p = 0.012; Scale 3: r = -0.531; p < 0.001; Scale 4: r = -0.424; p < 0.001) but only the scale 3 correlated to a weaker extent in TM (r = -0.275; p = 0.037).

**Table 3:** Univariate analysis of potential contributors for QoL.

		STAI-X	BDI	Chronic pain	PSQI	SSS							FBeK			
**Transwomen**																
						**ES**	**PS**	**SI**	**SB**	**RE**	**SA**	**APT**	**Attr.**	**Acc.**	**Unc.**	**Phys.**
SF-36	**CC**	-0.699	-0.626	-0.248	-0.622	0.147	0.245	0.373	-0.319	0.207	0.375	0.030	0.304	0.074	-0.531	-0.424
	**p**	**<0.001**	**<0.001**	**0.036**	**<0.001**	0.221	0.041	**0.001**	**0.008**	0.083	**0.001**	0.803	**0.012**	0.546	**<0.001**	**<0.001**
**Transmen**																
SF-36	**CC**	-0.453	-0.423	-0.355	-0.530	0.127	0.184	0.215	-0.324	0.111	0.149	0.073	0.213	-0.046	-0.275	-0.016
	**p**	**<0.001**	**<0.001**	**0.004**	**<0.001**	0.328	0.145	0.091	**0.009**	0.383	0.244	0.561	0.108	0.732	**0.037**	0.906

CC: Correlation coefficient (Pearson or Spearman as appropriate), SSS: Social Support Scale, ES: Emotional Support, PS: Practical Support,SI: Social Integration, SB: Social Burden, RE: Reciprocity, SA: Satisfaction, APT: Available Person of Trust; Attr: Attractiveness/self-confidence; Acc: Accentuation of physical appearance; Unc.: Uncertainty and anxiety in relation to the appearance; Phys: Physical reactions and physical–sexual discomfort

Bold numbers indicate significant differences

#### Linear regression analyses

To identify potential sex/gender-dependent predictors for QoL we carried out separate analyses for TM and TW. A three-block linear regression analysis was carried out to assess the contribution of different variables to the outcome QoL according to the SF-36 global score. Block 1 included BMI, age, level of education, hormonal therapy, estimation of the financial situation, having a partner, any genital surgery and unemployment. A forced entry method was used for block 1 as we deemed inclusion of these variables, according to earlier studies, a basic requirement for our model. In the second block, we added those variables that had been identified in univariate regression analysis as having a significant linear correlation with the SF-36 global score. Separate analyses were carried out for depressive symptoms (BDI, Block 2) and anxiety (STAIX; Block 3) due to strong correlations between those two variables (Pearson’s r = 0.654, p < 0.001 for TW and 0.524, p < 0.001 for TM).

#### Transmen

After full adjustment, the models explained for 22.8% (**Block 2**) and 19.7% (**Block 3**) of the total variance according to the adjusted R^2^ in TM (Table 4). In both models, the PSQI and the presence of chronic pain were the most significant predictors of QoL for TM (**Model 2;** PSQI global; β = -0.451; p = 0.003; chronic pain: β = -0.298; p = 0.042, **Model 3:** PSQI global β = -0.441; p = 0.002, chronic pain: β = -0.309; p = 0.031) (Table 4). Applying the models to all subscales of the SF-36 separately, it was shown that chronic pain was not only a predictor for the bodily pain domain in TM, but also significantly contributed to the physical functioning and physical role functioning domains, while the PSQI was an independent contributor for the subscales vitality, emotional role functioning and mental health (S1 Table).

**Table 4:** Determinants of QoL in Transmen.

Determinant variables	SF-36 global score			
Block 1	β	p (variable)	Adjusted R ^2^	p (model)
Age	-0.137	0.434	-0.119	0.934
BMI	0.068	0.719		
Hormonal therapy	-0.214	0.215		
Any genital surgery	0.016	0.932		
Being single	0.016	0.933		
Unemployment	0.029	0.879		
Financial situation	0.031	0.854		
Block 2*				
PSQI Global	-0.451	**0.003**	0.228	**0.039**
Chronic pain	-0.298	**0.042**		
Block 3**				
PSQI Global	-0.441	**0.002**	0.197	**0.040**
Chronic pain	-0.309	**0.031**		

* Block1 + PSQI global score, chronic pain, SSS Social burden score, FBeK Scale3+BDI

** +STAI instead of BDI

Bold numbers indicate significant differences

#### Transwomen

In TW, the fully adjusted models explained 51.9% (**Block 2**), respectively 65.0% (**Block 3**) of the variance of QoL according to the adjusted R^2^ (Table 5). In the model containing the BDI, the “best-fitting” variables were the PSQI global (β = -0.464; p < 0.001) the SSS subdomain social integration (β = 0.217; p = 0.049), FBeK Scale 3 (β = -0.434; p < 0.001) and being unemployed (β = -0.240; p = 0.034). The addition of the STAI-X global score (Block 3) instead of the BDI further improved the model (ΔR^2^ = 0.131). The regression models showed that neither in TW nor in TM the sociodemographic characteristics were useful in explaining the variance in the global score of the SF-36, although there were associations with some of its subdomains (S1 Table).

**Table 5:** Determinants of QoL in Transwomen.

Determinant variables	SF-36 global score			
Block 1	β	p (variable)	Adjusted R ^2^	p (model)
Age	-0.140	0.467	-0.089	0.902
BMI	0.152	0.403		
Hormonal therapy	-0.018	0.913		
Any genital surgery	0.159	0.645		
Being single	-0.124	0.430		
Unemployment	-0.064	0.693		
Financial situation	0.072	0.829		
Block 2**				
PSQI Global	-0.464	**< 0.001**	0.519	**< 0.001**
Social integration (SSS)	0.217	**0.049**		
FBeK Scale 3	-0.434	**< 0.001**		
Unemployment	-0.240	**0.034**		
Block 3**				
PSQI Global	-0.320	**0.002**	0.650	**< 0.001**
STAI total	-0.451	**< 0.001**		
Unemployment	-0.206	**0.020**		
FBeK Scale 3	-0.261	**0.011**		

FBeK Scale 3 = “Uncertainty and anxiety in relation to the appearance”

* Block 1 + PSQI global score, chronic pain, SSS social burden, Social integration, satisfaction, and FBeK Scale 1 and Scale 3, 4+BDI

** +STAI instead of BDI

Bold numbers indicate significant differences

#### Pain

**Fig 1:**
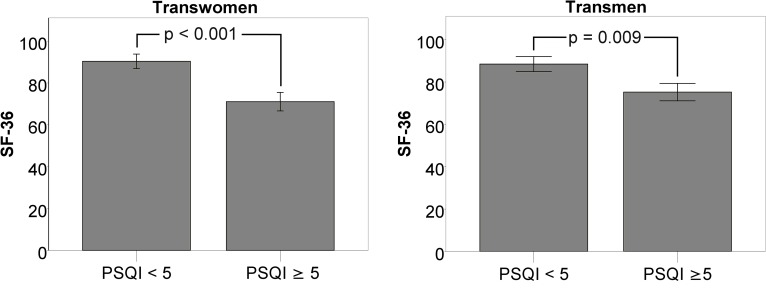
Effects of chronic pain on the SF-36 global score. The age-adjusted SF-36 global score was significantly lower (p = 0.006) in TM that reported to suffer from chronic pain, while this was only true on a trend level (p = 0.052) for TW.

As chronic pain was a significant independent predictor of QoL in our model, we were interested in the character of the reported symptoms related to chronic pain. Only the minority of patients was suffering from genital-related pain symptoms while the majority was suffering from pain associated with the musculoskeletal system (88.9% of TW vs. 70% of TM; n.s.), mainly from any kind of back pain. 50% of TW and 40% of TM in this subgroup reported on any kind of chronic headaches (S3 Table). In accordance with the results from the regression models, TM with chronic pain had a significantly lower SF-36 score than those without (70.4 ±13.8 vs. 81.0 ±12.3; p = 0.006), while this difference was shown in TW only on trend-level (p = 0.052) (Fig 1).

#### Sleep

**Fig 2:**
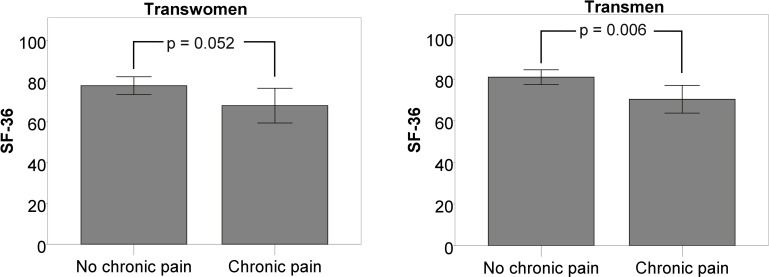
Effects of poor sleep on the SF-36 global score. The age-adjusted SF-36 global score was significantly lower in TM (p = 0.009) and TW (p < 0.001) that reported to suffer from poor sleep indicated by a PSQI global score of ≥ 5.

Those with a PSQI ≥5, indicating poor sleep, had a significantly lower SF-36 score than those with good sleep in both groups (71.1±16.4 vs. 90.4±6.9 in TM; 75.2±15.0 vs. 88.5±6.2 in TW, p = 0.009, <0.001 respectively) (Fig 2). As nightly sweating and flushing had been suggested of being a significant contributor to sex steroid-dependent sleep disturbances, especially in postmenopausal women from the general population, we also analyzed data that were available from our sample on flushing and sweating.

Flushing as well as sweating at night and at rest was more common in TM than in TW (S2 Table). Sweating and flushing did not differ according to SHT status but nightly sweating was more common in TM that had undergone gonadectomy than in those without (p = 0.009; data not shown). Additionally, nightly sweating was positively correlated in a univariate analysis with the PSQI as an indicator of poorer sleep in TM but not TW (r = 0.433; p < 0.001). Only one transwoman and no TM were suffering from a diagnosed obstructive sleep apnea syndrome (OSAS) and none from restless leg syndrome that had also been associated with sex-steroid-dependent poor sleep before (data not shown).

## Discussion

The aim of the present study was to identify sex/gender-specific factors that influence health-related QoL in individuals with GD. With this approach, we are aiming to detect potential targets for therapeutic interventions to ultimately improve QoL in these patients. Interestingly, the factor best explaining QoL in our sample was impaired sleep quality, resulting in a significant negative impact on QoL in both sexes.

In contrast, the presence of chronic pain symptoms affected QoL in TM only. Importantly, there was no significant difference with regard to the prevalence of pain between the sexes. Modelling QoL in TM also seems to be more complex. At least, our model could only explain for 22.8% of the variability of the SF-36 while it could explain up to 65% of the SF-36 in TW.

We furthermore identified that being unemployed as well as "self-perception with regard to the body image" in terms of "uncertainty and anxiety in relation to the appearance" in the FBeK score were significant independent determinants of the SF-36 in TW but not in TM. "Satisfaction with the body image" was shown before to be an independent predictor of QoL in the general population [32]. Thus, we found gender associated commonalities but also differences.

### Social support

Perceived social support may affect QoL by different mechanisms such as reducing experienced stress, increasing personal resources or by supporting active coping strategies [33]. In our cohort, TW had lower scores for the social support questionnaire subdomains “practical support”, “social integration” and “satisfaction”, indicating a general lower perceived social support in comparison to TM. It has been shown before that TW have lower scores in perceived social support scales compared with natal women [34]. However, in the general population perceived social support is usually reported to be higher for women than for men [35]. This discrepancy could possibly be explained by the high degree of discrimination in various domains of live and less social support and acceptance by family members in TW in comparison to TM. It has been demonstrated before that the inverse relationship between perceived stigmatization and psychological well-being is mediated by social support [36]. All three social support domains were significantly correlated with QoL in TW, while this was only true for the domain “social burden” in TM. It has been demonstrated before hat social support can be an independent predictor of QoL in TW [37], as well as in TM [38]. It has also been shown that social support seems to be more important for general and psychosocial well-being in women than in men [39].

The subdomain "social integration" was a significant positive contributor to the SF-36 global score in TW but not in TM. The domain social integration gives information on the availability of friends and/or persons sharing similar interests to conduct joint activities. Peer groups have been considered a significant source of social support, providing access to new coping strategies, sharing similar experiences or strengthening the feeling of belonging to a group that may subsequently improve emotional well-being and QoL [40]. The relationship of the “social integration” score with QoL was independent of depressive symptoms according to the BDI-score, but was no longer significant after adjustment for the STAIX score, indicating a mediating effect of anxiety traits. It has been shown before that social support is strongly negatively correlated with the perception of anxiety symptoms in TW [37].

In TM the only domain of social support influencing QoL was “social burden”, which correlated inversely with the subdomain "social role functioning" of the SF-36. This association was still significant in the fully adjusted model for this subdomain. The "social burden" score refers to the perceived feeling of e.g. being criticized or rejected by others.

### The impact of sleep on QoL

The rate of reported poor sleep in our sample was unexpectedly high with around 80% of TM and TW having a PSQI global score equal to or above 5, which indicates poor sleep quality. Strine and colleagues did show that experienced sleep quality has a huge impact on various aspects of general well-being and is a significant predictor of QoL in the general population [41]. This is of importance, because sleep quality as well as sleep duration has moreover been shown to be a predictor of morbidity [42] and mortality [43] in several studies. A recent epidemiological study from Germany reported the prevalence of poor sleep according to the PSQI to be 62.5% for women and 37.5% for men [44] and it is a common finding that more women report poor sleep than men [41]. However, we did not observe any sex-dependent differences in our sample. Thus, the ratio between female and male subjects regarding problems with sleeping seems to be disrupted because TW and TM reported high levels of poor sleep.

The causes for poor sleep did not seem to be solely explained by the degree of chronic pain or mood disturbances which are commonly known to influence sleep quality [45]. We can only speculate on the effect of SHT in this context. The finding that neither mean PSQI scores nor the number of those classified as good or poor sleepers did differ between those with and those without SHT (data not shown), speaks against a significant role of sex hormones in this context. However, we have to acknowledge that the sample size of treatment-naïve patients was rather small. In general, sex hormones are known to affect sleep [46] and our group has shown that SHT in TW can affect sleep architecture [47]. The mostly studied population with regard to the effects of sex steroids on sleep are hypogonadal or postmenopausal women. In these patients, estrogen replacement therapy generally improves sleep e.g. by decreasing sleep latency and nightly wakefulness [46, 48]. Apart from sex steroids affecting sleep architecture on a central level, a mechanistically more simple explanation in this context has been the beneficial effects of estradiol on flushing and night sweats. Although all our patients were regarded by their treating physicians as being on adequate SHT and therefore should not suffer from hypogonadal symptoms, we also had a look on reported flushing and sweating in our cohort. Interestingly, sweating at night and flushing was more common in TM than in TW. This was also associated with poorer sleep in TM but not TW. Therefore we might speculate that the observed high prevalence of poor sleep may actually be driven by different mechanisms in both sexes.

Testosterone can as well effect sleep. In natal men, low testosterone levels may lead to poor sleep, which improves after replacement therapy. The role of testosterone in natal women is not fully understood. It has been shown repeatedly that women with polycystic ovarian syndrome (PCOS) and hyperandrogenism suffer from impaired sleep, possibly being related to sleep-disordered breathing or a high BMI [49]. According to self-report and the medical files, only one transwoman was diagnosed with OSAS. However, we do so far not systematically screen for this condition in our patients, though it is a known side effects of testosterone replacement therapy in hypogonadal men.

Lastly, progesterone is also known to have effects on sleep by inducing sleep onset in both sexes [50]. It remains speculative though if a progesterone “deficit” in TM contributes to the observed sleep disturbances.

Taken together, insomnia in transgender people is an independent influencing factor on quality of life assessed with the SF-36. The underlying mechanisms thereon need to be elucidated with further studies that preferably investigate sleep quality in a longitudinal manner before and after initiation of SHT. Given the known impact of gonadal hormones on sleep architecture, quantity and quality, further clinical studies on the influence of these hormones on sleep seem to be promising.

### Pain

It has been shown before that pain is a significant determinant of QoL in the general population [51] and may also significantly influence different subscales of the SF-36 [52]. Potential sex differences have only rarely been investigated in detail [45]. Bingefors and colleagues [53] did show that there are not only differences in the prevalence of pain but also sex differences with regard to how pain is affecting QoL. They demonstrated that headache had more impact on the physical dimensions of QoL among men; while the psychological dimensions were more affected among women [53].

We could show that chronic pain was an independent predictor for QoL in TM but not in TW, although the pain prevalence of about 20% did not differ between the sexes. In the general population, the prevalence of chronic pain is highly variable between studies and ranges from 7% [54] to 55% [55]. However, consistently almost all epidemiological studies show a higher prevalence of most pain conditions for women in comparison to men [56].

There are few studies [57] dealing with pain in transgender people aside from those investigating pain in the specific context of TRS [58]. Interestingly, our study did show that only a minority of patients in both groups did suffer from any genital related pain symptoms. Most were affected by headaches and musculoskeletal pain. These are the types of pain that are most commonly found in the general population, too [53]. In particular, headache is reported to be up to 3-times higher in women than in men followed by back pain with an OR of 1.2 [56, 59].

Alosi et al. did show that 29.8% of TW and 61.5% of TM reported any kind of pain. In these two groups headaches and musculoskeletal pain were the most common pain conditions, too. By retrospective assessment, the authors showed that initiation of SHT was associated with a higher incidence of pain in TW while the opposite was true for TM. The authors suggested that this could be a direct effect of the changed sex hormonal milieu [57] and explain for sex differences in pain reporting in the general population. However, the reason for the observed sex-dependent discrepancies in pain frequency including direct effects of sex steroids on pain perception or differences with regard to reporting behavior are still a matter of debate [60]. Controversially, Motsman and colleagues did show that TM show lower scores in bodily pain than TW [10].

### The psychological impact on QoL

Not surprisingly, our analysis showed a strong inverse correlation of depressive symptoms (BDI) and QoL. Of note, the sample comprised 10 participants with moderate (5 TW, 5 TM) and 4 participants (3 TW, 1 TM) with severe depressive symptoms. Importantly, the regression model revealed that poor sleep had a strong and independent influence on QoL in both sexes and was moreover associated with several domains of the SF-36, such as emotional role functioning and vitality. Chronic pain influenced QoL in TM only, while anxiety showed an independent influence on QoL in TW. These BDI-independent effects on the SF-36 are of great importance because insomnia, perception of pain as well as anxiety are symptoms of depression as well. The present study, however, shows, that these symptoms influence QoL assessed with the SF-36 independent of depressive symptoms.

Importantly, sleep [61] as well as pain-perception [55] are highly influenced by psychosocial stress [61]. This could be of great importance in this sample because transgender individuals seeking transition-related health care often suffer from psychosocial stress both due to being stigmatized as a sex/gender minority and due to their gender dysphoria per se [62]. As a matter of fact mental health care can improve not only sleep but as well pain perception. This has been demonstrated for cognitive behavioral therapy addressing insomnia and lower back pain [63]. Moreover, these interventions can improve QoL [64].

Taken together, psychobiological models of insomnia [53] and pain [65] may be of great importance to understand health related QoL in GD individuals. However, the interplay between psychological stressors, physical and endocrinological alterations on sleep and pain in people with GI/GD await further investigations.

### Limitations

The concept of QoL is highly complex and includes a variety of potentially influencing factors, which can differ depending on the population studied. Due to the low prevalence of GD the number of subjects studied so far is limited. Therefore, it is difficult to include all factors that could potentially influence QoL into our analysis. We therefore focused on variables that have been shown to have a significant impact on Qol in these subjects and on those representing psychological well-being such as the BDI and STAIX. Our model could explain for up to 65% of the variability of the SF-36 in TW, but only for 22.8% in TM. This further shows that there is still need for the identification of more relevant and potentially modifiable factors contributing to QoL in this special cohort, especially in TM.

## Conclusion

In conclusion, our findings of sex-dependent determinants of QoL in transgender individuals seeking transition-related health care may have implications for the long-term management of this target group. The knowledge that a substantial proportion of the reduced QoL is due to the high incidence of the so far under-studied poor sleep in both sexes, anxiety in TW and chronic pain in TM emphasizes the need for a diagnostic work-up including these parameters with the ultimate goal to find sufficient strategies for a holistic transgender health care approach and improve QoL in the long run.

## Supporting information

S1 Table Sex-dependent regresssional analysis of determinants on SF-36 subdomains. (XLSX)

S2 Table Pain characteristics. (DOCX)

S3 Table Sweating and flushing. (DOCX)
