# Supplementary material for: High impact of sleeping problems on quality of life in transgender individuals: A cross-sectional multicenter study
Source: PLoS One. 2017 Feb 15;12(2):e0171640. doi: 10.1371/journal.pone.0171640 (PMC5310898; doi:10.1371/journal.pone.0171640)
Supplement: S2 Table — (DOCX) [file pone.0171640.s002.docx]

**S2 Table Pain characteristics**

|  | **Transwomen** | | **Transmen** | |  |
| --- | --- | --- | --- | --- | --- |
|  | **N** | **%** | **N** | **%** | **p** |
| Any chronic pain | 18 |  | 20 |  | n.s. |
| Musculoskeletal | 16 | 88.9 | 14 | 70 | n.s. |
| Back pain | 12 | 66.7 | 9 | 45 | n.s. |
| Headaches | 9 | 50.0 | 8 | 40 | n.s. |
| Genitals | 5 | 27.8 | 3 | 15 | n.s. |
| Breast | 0 | 0.0 | 1 | 5 | n.s. |
| Whole body | 0 | 0.0 | 2 | 10 | n.s. |
| Other | 1 | 5.6 | 0 | 0 | n.s. |
| Not specified | 0 | 0.0 | 4 | 20 | **0.045** |

Bold numbers indicate significant differences
