# Supplementary material for: High impact of sleeping problems on quality of life in transgender individuals: A cross-sectional multicenter study
Source: PLoS One. 2017 Feb 15;12(2):e0171640. doi: 10.1371/journal.pone.0171640 (PMC5310898; doi:10.1371/journal.pone.0171640)
Supplement: S3 Table — (DOCX) [file pone.0171640.s003.docx]

**S3 Table Sweating and flushing**

|  | **Transwomen** | | | **Transmen** | | |  |
| --- | --- | --- | --- | --- | --- | --- | --- |
|  | **Median** | **Min** | **Max** | **Median** | **Min** | **Max** | **p** |
|  |  |  |  |  |  |  |  |
| Flushing | 0 | 0 | 4 | 0.5 | 0 | 5 | **0.04** |
| Sweating at night | 1 | 0 | 5 | 2 | 0 | 5 | **0.021** |
| Sweating at rest | 0 | 0 | 3 | 0 | 0 | 5 | **0.005** |
| Sweating during exertion | 0 | 0 | 4 | 0 | 0 | 4 | 0.332 |

Compared by Mann-Whitney-U Test

Bold numbers indicate significant differences

TM did significantly more often report on suffering from flushing, sweating at rest as well as at night
